# Horizontal inequity trends of health care utilization in rural China after the medicine and healthcare system reform: based on longitudinal data from 2010 to 2018

**DOI:** 10.1186/s12939-023-01908-4

**Published:** 2023-05-17

**Authors:** Jinpeng Xu, Guomei Tian, Jiale Sun, Jian Liu, Fangting Chen, Qi Shi, Ting Zhang, Hongyu Zhang, Jingran He, Fangmin Deng, Bokai Zhang, Haixin Wang, Qunhong Wu, Zheng Kang

**Affiliations:** 1 School of Health Management, Harbin Medical University, Harbin, China; 2 Department of Nuclear Medicine, The Fourth Hospital of Harbin Medical University, Harbin, China; 3 Xuzhou Center for Disease Control and Prevention, Xuzhou, China

**Keywords:** Health care utilization, Concentration index, Horizontal inequity, Rural China

## Abstract

**Background:**

To assess the effectiveness of China's medicine and health care reform in promoting equity in health care utilization among rural residents, it is necessary to analyze temporal trends in equity in health care utilization among rural residents in China. This study is the first to assess horizontal inequity trends in health care utilization among rural Chinese residents from 2010 to 2018 and provides evidence for improving government health policies.

**Methods:**

Longitudinal data obtained from China Family Panel Studies from 2010 to 2018 were used to determine trends in outpatient and inpatient utilization. Concentration index, concentration curve, and horizontal inequity index were calculated to measure inequalities. Decomposition analysis was applied to measure the contribution of need and non-need factors to the unfairness.

**Results:**

From 2010 to 2018, outpatient utilization among rural residents increased by 35.10%, while inpatient utilization increased by 80.68%. Concentration indices for health care utilization were negative in all years. In 2012, there was an increase in the concentration index for outpatient utilization (CI = -0.0219). The concentration index for inpatient utilization decreased from -0.0478 in 2010 to -0.0888 in 2018. Except for outpatient utilization in 2012 (HI = 0.0214), horizontal inequity indices for outpatient utilization were negative in all years. The horizontal inequity index for inpatient utilization was highest in 2010 (HI = -0.0068) and lowest in 2018 (HI = -0.0303). The contribution of need factors to the inequity exceeded 50% in all years.

**Conclusions:**

Between 2010 and 2018, low-income groups in rural China used more health services. This seemingly pro-poor income-related inequality was due in large part to the greater health care need among low-income groups. Government policies aimed at increasing access to health services, particularly primary health care had helped to make health care utilization in rural China more equitable. It is necessary to design better health policies for disadvantaged groups to reduce future inequities in the use of health services by rural populations.

## Introduction

Equity in access and utilization of health care is an important goal for health systems worldwide [1, 2], which is also an important indicator of the overall health performance of a region or country. However, inequalities in the use of health care are prevalent across regions and countries, particularly pronounced among people at different income levels [3–5]. Inequalities in health care utilization significantly undermine the health and well-being of populations, especially low-income groups, who are more likely to be unhealthy and have a greater need for health services. However, due to various factors such as low education or lack of health insurance, they may use fewer health services than the wealthy [6], and thus their health status may further deteriorate. Inequitable health care utilization is not only detrimental to the enjoyment of basic health rights by low-income individuals [7], but also significantly contributes to health inequities in the population [8].

Previous research has provided a rich foundation for research on health care utilization inequity. Masseria and Doorslaer [9] analyzed income-related inequalities in health care utilization in 21 OECD countries. They found that approximately half of the countries had horizontal inequalities in the probability of visiting a doctor and the number of visits in favor of the rich. Between 1998 and 2008, health care utilization in Brazil became increasingly equitable, shifting from a largely favorable utilization of health care services by the rich to a slightly excessive utilization by the rich [10]. Similarly, outpatient utilization and inpatient utilization in China vary among individuals with different incomes [11–13]. According to the China National Health Services Survey, the concentration index for outpatient and inpatient health service utilization in China in 2008 was 0.015 and 0.197, respectively, with higher-income groups using more outpatient and inpatient services [14]. This phenomenon is also present in outpatient and inpatient services for middle-aged and elderly groups [15], dental care for preschool children [16], and maternal health services for rural women [17].

Existing studies have found that the equity of health care needs and utilization among rural residents is poor compared to urban areas [18, 19]. Disparities in health care utilization exist among rural residents of different economic levels [20]. In 2009, China systematically implemented a comprehensive reform of its medicine and healthcare system, which insisted on a rural focus and centered on establishing a national primary healthcare system and strengthening insurance programs for low-income citizens (now at almost 100%), thus achieving universal health coverage. The reform has improved access to basic medical services for rural residents and has played a huge role in promoting the utilization of health services for rural residents. Yan, Liu [21] showed that rural residents' outpatient and inpatient utilization increased significantly between 2008 and 2018, with outpatient utilization increasing from 15.5% to 25.2% and inpatient utilization increasing from 6.9% to 14.3%. However, health care reform should consider the goal of equality as a priority objective [22], and it is necessary to assess the impact of the reform on the equity of health care utilization of the residents. To the best of our knowledge, fewer studies have explored the equity of health care utilization among rural Chinese residents at a national level after China's deepening medical and healthcare system reform. Most existing studies have conducted cross-sectional studies using data from a single period in a given year [23, 24] or simply assessing the effects of the implementation of a particular policy of the reform [25, 26], making it difficult to capture the continuous impact of policy changes of the reform on the equity of health care utilization in rural China.

To assess the effectiveness of China's medical and healthcare reforms in promoting equity in health care utilization among rural residents, it is necessary to conduct a longitudinal analysis of temporal trends in equity in rural health care utilization. Therefore, the purpose of this study was to *i)* assess income-related inequalities and horizontal inequalities in the use of health services in rural China and their changing trends during the reform of China’s medicine and healthcare system and *ii)* explore the contribution of need and non-need factors to the observed inequities.

## Methods

### Data source

We adopted longitudinal data from a series of cross-sectional household surveys known as the China Family Panel Study (CFPS). CFPS is a national longitudinal project that began in 2010, and the sample covers 25 provinces in China excluding Hong Kong, Macau, Taiwan, Xinjiang, Qinghai, Inner Mongolia, Ningxia, and Hainan, which account for 95% of the total population of China. Therefore, the CFPS sample can be considered a nationally representative sample.

The sampling method of CFPS is based on the multistage approach using the implicit stratification method [27], where the sample is drawn through a three-stage sample. The first stage sample is administrative districts/counties, the second stage sample is administrative villages/residential committees, and the third stage sample is household households. In the first two stages, the sampling used official administrative district information, while in the third stage, the sampling frame was constructed using the map address method, and the sample households were drawn using circular equidistant sampling with a random starting point.

The CFPS program collects data every two years, and the target sample size for the 2010 follow-up survey is 16,000 households, which includes all household members in the sample. The objective is to investigate families’ and individuals’ information on a range of topics, including economic status, state of health, and access to and utilization of health care services. Considering the impact of COVID-19 on health care utilization in 2020, we used the data from five periods collected from 2010 to 2018. This study selected rural residents as the participants. After removing cases with missing values, a total of 74,773 person-times were obtained as valid samples.

### Variable definition

In this study, outcome variables were measured by health care utilization in outpatient and inpatient service, derived from the questions: “Have you seen a doctor during the past two weeks?” and “In the past year, were you ever been hospitalized due to illness?”. We used the same questions to measure health care utilization among rural residents, thus ensuring that the measurements are longitudinally comparable.

To measure the horizontal inequity in health care utilization, it is necessary to standardize individuals’ health care needs. The standardized health care need refers to the forecast of health care needs generated by one’s health status under the control of socioeconomic factors such as income and education level. We considered multiple need and non-need determinants of health care utilization, using the approach adopted by Newbold, Eyles [28] and Jones, Abásolo [29]. Based on guidelines developed by World Bank [30], control variables were set to represent health care need and other non-need factors.

Each individual’s health care need was approximated by demographic (age and gender) and health (self-rated health and chronic diseases) variables. Age was categorized into three groups: less than 30 years, 30–59 years, and 60 years and older. Gender was defined as male and female. The self-rated health status variable was grouped into three groups: poor, fair, and good. The information for chronic diseases was derived from the question: “During the past six months, have you had any doctor-diagnosed chronic disease?”.

Non-need variables included education level, marital status, medical insurance, employment status, and socioeconomic status variables. Education level was categorized into four groups: primary school or below, junior high school, high/secondary school, and college or above. Marital status was dichotomized into married or single, and single included those who were unmarried, divorced, and widowed. Medical insurance variable and employment status were defined as yes or no. Socioeconomic status (SES) was measured by the per capita annual household income of the participants. For all multi-category variables, dummy variables were created for all categories, using the highest category as the reference group. Note that we do not adjust per capita annual household income for inflation, since we divided individuals based on “rank” for the quintiles included in the decomposition. This means that in the statistical analysis, income is treated as a relative measure of SES in each period.

### Statistical analysis

In this study, concentration index (CI), concentration curve (CC), and horizontal inequity index (HI) were calculated to measure inequality in health care utilization. CI values range from − 1 to + 1. The positive (negative) value indicates that health care utilization is concentrated among rich (poor) individuals. CI equals zero means there is no inequality [31].

1 $$C=\frac{2}{\mu }cov({y}_{it},{r}_{it})$$

where C is the concentration index, μ is the mean of health care utilization, *cov* is the covariance, *y*_*i*_ is the health variable, *r*_*i*_ is the *i*^*th*^ individual’s fractional rank in the SES distribution and *t* is the time variable.

The results of Goddard and Smith’s [32] highlight the importance of health care need adjustment. Therefore, we employed measures of horizontal inequity developed by Wagstaff, van Doorslaer [33] and van Doorslaer, Wagstaff [34]. HI is defined as the difference between observed health care utilization and that which would be expected given the individual’s health care needs. Taking into account the fact that individuals have different health care needs and that differences in health care needs ought to translate into different needs for and use of health services. Once health care needs are standardized across individuals, remaining utilization could be considered to be inequitable. Therefore, we need to control for the impact of individual needs on the use of health services. When residents have the same need for health care, whether people can enjoy fair health services is the horizontal inequity [35].

Based on the characteristics of the data in this study, we used an indirect method to estimate the standardized health care needs of individuals [36]. Since the outcome variables in this study are both binary categorical variables, we used the linear approximation of a probit model to estimate partial effects [37]:

2 $${y}_{i}={\alpha }^{m}+{\sum }_{j}{\beta }_{j}^{m}{x}_{ji}+{\sum }_{k}{\gamma }_{k}^{m}{z}_{ki}+{\varepsilon }_{i}$$

where *y*_*i*_ is the use of the particular health care by individual *i*, that is, the expected health care use of individual *i* based on his/her health care needs. *x*_*j*_ is a vegetation of need factors, *z*_*k*_ is a vegetation of non-need factors, ${\beta }_{j}^{m}$ and ${\gamma }_{k}^{m}$ are the partial effects (*dy/dx*_*j*_, *dy/dz*_*k*_) for *x*_*j*_ and *z*_*k*_, and ε is the error term.

Decomposition analysis can determine the contribution of each influencing factor to the unfairness related to SES. The contribution of each influencing factor is equal to the product of the sensitivity of each factor to the dependent variable and the concentration index of each factor. The decomposition of the concentration index can thus be expressed as the following formula:

3 $$C=\sum (\frac{{\beta }_{j}^{m}{\overline{x} }_{j}}{\mu }){C}_{j}+\sum \left(\frac{{\gamma }_{k}^{m}{\overline{z} }_{k}}{\mu }\right){C}_{k}+\frac{G{C}_{\varepsilon }}{\mu }$$

In formula (3), ${\overline{x} }_{j}$,${\overline{z} }_{k}$ and μ are the mean levels of *x*_*j*_, *z*_*k*_ and *y*_*i,*_ respectively. $(\frac{{\beta }_{j}^{m}{\overline{x} }_{j}}{\mu }){C}_{j}$ and $\left(\frac{{\gamma }_{k}^{m}{\overline{z} }_{k}}{\mu }\right){C}_{k}$ are the contributions of need variables and non-need variables. $\frac{G{C}_{\varepsilon }}{\mu }$ is the generalized concentration index for the remaining error [38].

Once the concentration indices for actual and predicted needs are calculated, HI is calculated by formula (4). A positive HI represents that the health service needs of the higher-income population are better met and vice versa. Zero represents complete equity, that is, the same health service needs are met equally.

4 $$HI=C-\sum \left(\frac{{\beta }_{j}^{m}{\overline{x} }_{j}}{\mu }\right){C}_{j}=\sum \left(\frac{{\gamma }_{k}^{m}{\overline{z} }_{k}}{\mu }\right){C}_{k}+\frac{G{C}_{\varepsilon }}{\mu }$$

Stata 16.0 (Stata Corp, College Station, TX, USA) was used for data cleaning and preprocessing. A two-tailed *P*-value less than 0.05 was considered statistically significant.

## Results

### Descriptive statistics

Table 1 presents the characteristics of the study sample in each survey year. While the gender distribution had not changed much over the five survey periods, the proportion of elderly individuals in the survey population had shown an increasing trend year by year. The proportion of people aged 60 and over rose by 8.71%. The education level of the survey population also increased. Between 2010 and 2018, there was a 6.49% decrease in those with primary school education or below.

**Table 1:** Description of sample distribution in rural China, 2010–2018 (%)

Characteristic	2010(*N* = 16,162)	2012(*N* = 15,120)	2014(*N* = 14,656)	2016(*N* = 14,966)	2018(*N* = 13,869)
**Health care utilization**					
Outpatient utilization	3330(20.60)	3333(22.04)	3609(24.62)	3623(24.21)	3860(27.83)
Inpatient utilization	1288(7.97)	1389(9.19)	1679(11.46)	1792(11.97)	1997(14.40)
**Gender**					
Male	7993(49.46)	7490(49.54)	7276(49.65)	7554(50.47)	6947(50.09)
Female	8169(50.54)	7630(50.46)	7380(50.35)	7412(49.53)	6922(49.91)
**Age**					
< 30	3123(19.32)	2712(17.94)	2538(17.32)	2557(17.09)	1921(13.85)
30 ~ 59	9756(60.36)	8934(59.09)	8349(56.97)	8366(55.90)	7923(57.13)
≥ 60	3283(20.31)	3474(22.98)	3769(25.72)	4043(27.01)	4025(29.02)
**Self-rated health**					
Poor	3243(20.07)	3376(22.33)	2737(18.67)	2787(18.62)	2832(20.42)
Fair	5344(33.07)	2662(17.61)	2058(14.04)	2607(17.42)	1832(13.21)
Good	7575(46.87)	9082(60.07)	9861(67.28)	9572(63.96)	9205(66.37)
**Chronic disease**					
Yes	2401(14.86)	1868(12.35)	2483(16.94)	2550(17.04)	2524(18.20)
No	13,761(85.14)	13,252(87.65)	12,173(83.06)	12,416(82.96)	11,345(81.80)
**Education level**					
Primary school or below	10,260(63.48)	9815(64.91)	9243(63.07)	9084(60.70)	7904(56.99)
Junior high school	4285(26.51)	3646(24.11)	3765(25.69)	3883(25.95)	3891(28.06)
High/Secondary school	1354(8.38)	1239(8.19)	1215(8.29)	1383(9.24)	1393(10.04)
College or above	263(1.63)	420(2.78)	433(2.95)	616(4.12)	681(4.91)
**Marital status**					
Married	13,095(81.02)	12,788(84.58)	12,370(84.4)	12,576(84.03)	11,693(84.31)
Single	3067(18.98)	2332(15.42)	2286(15.60)	2390(15.97)	2176(15.69)
**Medical insurance**					
Yes	13,896(85.98)	13,764(91.03)	13,669(93.27)	13,874(92.70)	12,855(92.69)
No	2266(14.02)	1356(8.97)	987(6.73)	1092(7.30)	1014(7.31)
**Employment status**					
Employed	8737(54.06)	8641(57.15)	11,775(80.34)	12,066(80.62)	11,292(81.42)
Unemployed	7425(45.94)	6479(42.85)	2881(19.66)	2900(19.38)	2577(18.58)
**SES**					
Poorest SES	3234(20.01)	3025(20.01)	2934(20.02)	3011(20.12)	2782(20.06)
2th SES	3231(19.99)	3025(20.01)	2940(20.06)	2977(19.89)	2768(19.96)
Middle SES	3233(20.00)	3024(20.00)	2921(19.93)	3021(20.19)	2772(19.99)
4th SES	3232(20.00)	3028(20.03)	2930(19.99)	2967(19.82)	2784(20.07)
Highest SES	3232(20.00)	3018(19.96)	2931(20.00)	2990(19.98)	2763(19.92)

**Fig. 1:**
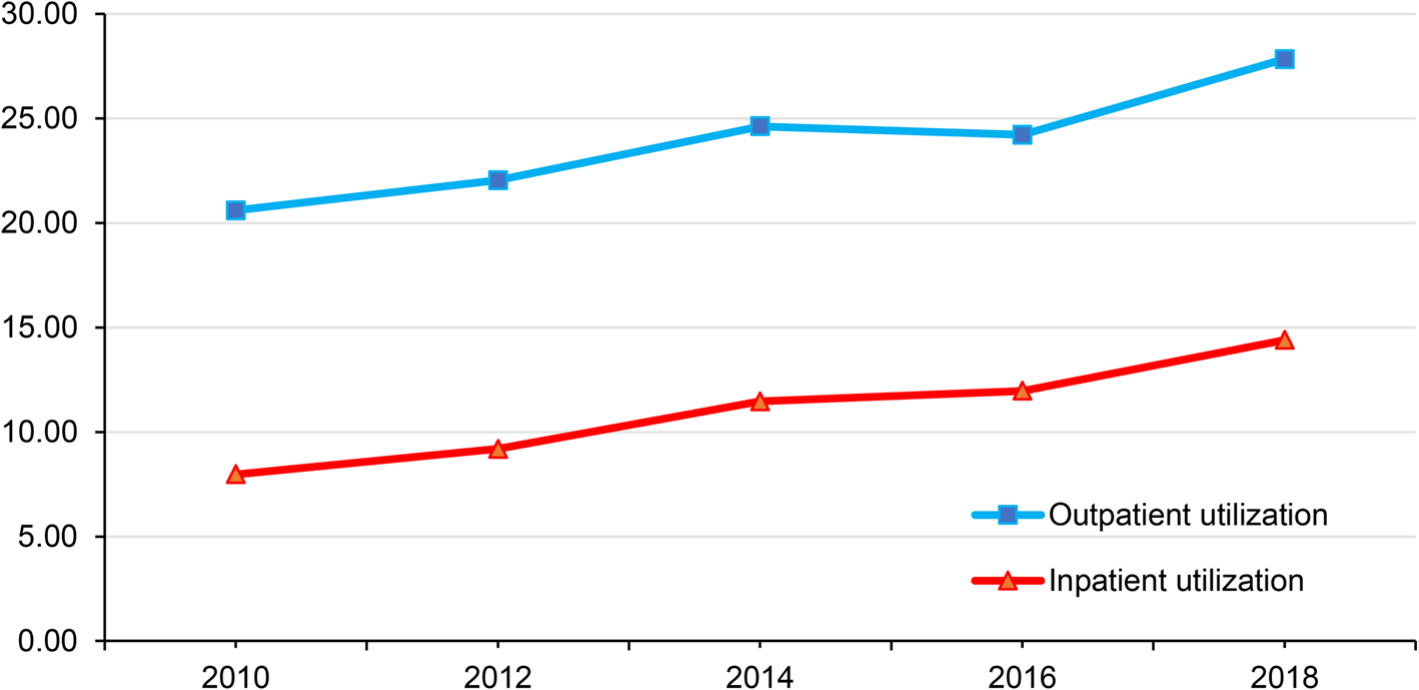
Utilization of health care among rural residents, 2010–2018

In terms of health care utilization, as shown in Fig. 1, although the inpatient utilization for rural residents fluctuated between 2014 and 2016, overall, throughout the five periods of the survey, health care utilization for rural residents continued to show an upward trend, with outpatient utilization increasing from 20.60% in 2010 to 27.83% in 2018 (% change = 35.10%) and inpatient utilization increasing from 7.97% in 2010 to 14.40% in 2020 (% change = 80.68%).

### Inequality and horizontal inequity for health care utilization

Table 2 shows the CI and HI for health care utilization in rural China, 2010–2018. Concentration indices for all five surveys were negative for both outpatient and inpatient utilization, with the CI for outpatient utilization in 2012 (CI = -0.0219) and inpatient utilization in 2010 (CI = -0.0478) being closest to zero. The magnitude of CI changes varied over time, with the pro-poor propensity of rural residents to utilize outpatient care increasing by 19.59% in 2018 compared to 2010, and the pro-poor propensity of inpatient care increasing by 85.77%.

**Table 2:** Unstandardized Concentration Index and Horizontal inequity index, 2010–2018

	**Health care utilization**	**CI**	**HI**
2010	Outpatient utilization	-0.0735	-0.0272
	Inpatient utilization	-0.0478	-0.0068
2012	Outpatient utilization	-0.0219	0.0214
	Inpatient utilization	-0.0518	-0.0235
2014	Outpatient utilization	-0.0743	-0.0327
	Inpatient utilization	-0.0618	-0.0264
2016	Outpatient utilization	-0.0735	-0.0213
	Inpatient utilization	-0.0692	-0.0122
2018	Outpatient utilization	-0.0879	-0.0287
	Inpatient utilization	-0.0888	-0.0303

**Fig. 2:**
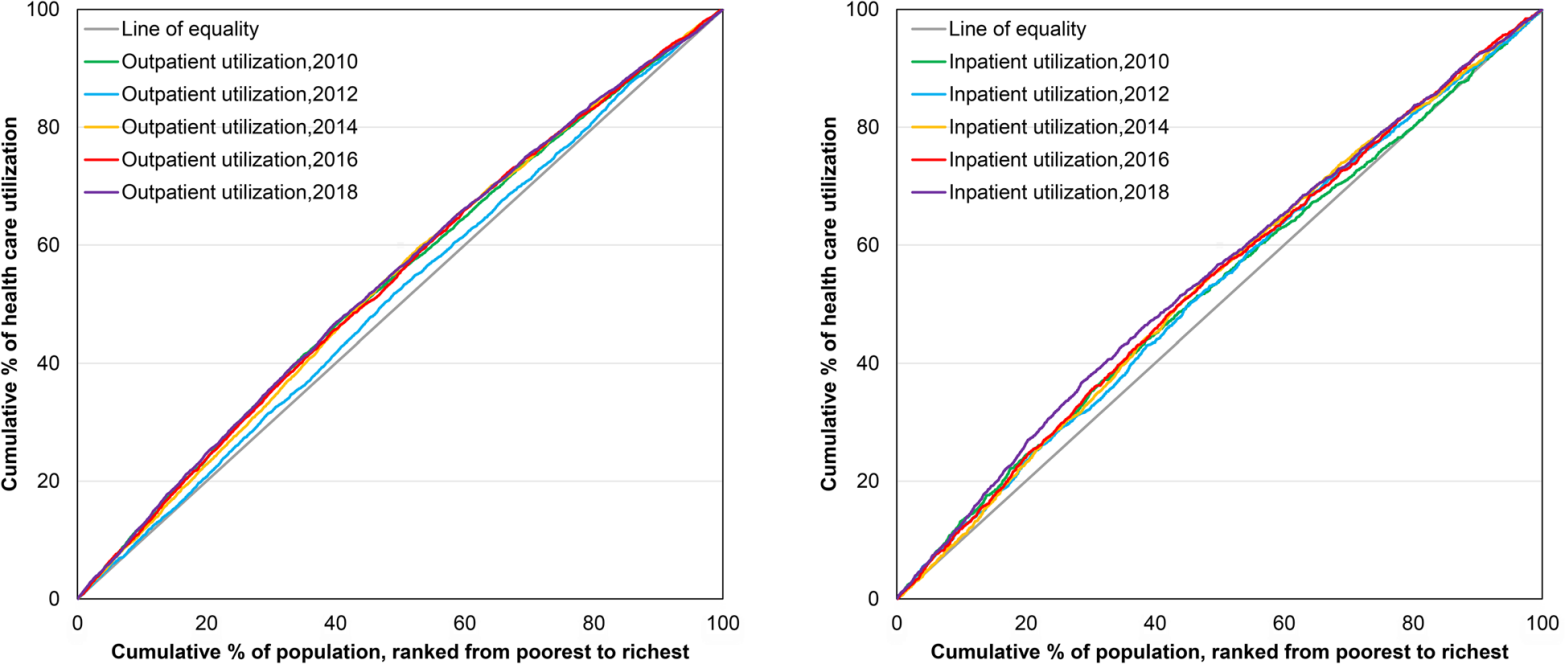
Concentration curves on health care utilization, 2010–2018

Figure 2 displays concentration curves that illustrate the unadjusted concentration indices presented in Table 2 for health care utilization from 2010 to 2018. From Fig. 2, we can clearly observe that the CC for all health services is below the equality line, regardless of the period, confirming what was indicated by the CI. At the same time, we can see the trend in CI over time. In terms of outpatient utilization, the change in CI was not significant for the rest of the years, except for 2012, when there was a significant rise in inequity (but still favored the poor), while from 2010–2018, the inequity in inpatient utilization for rural residents showed a year-on-year increase.

**Fig. 3:**
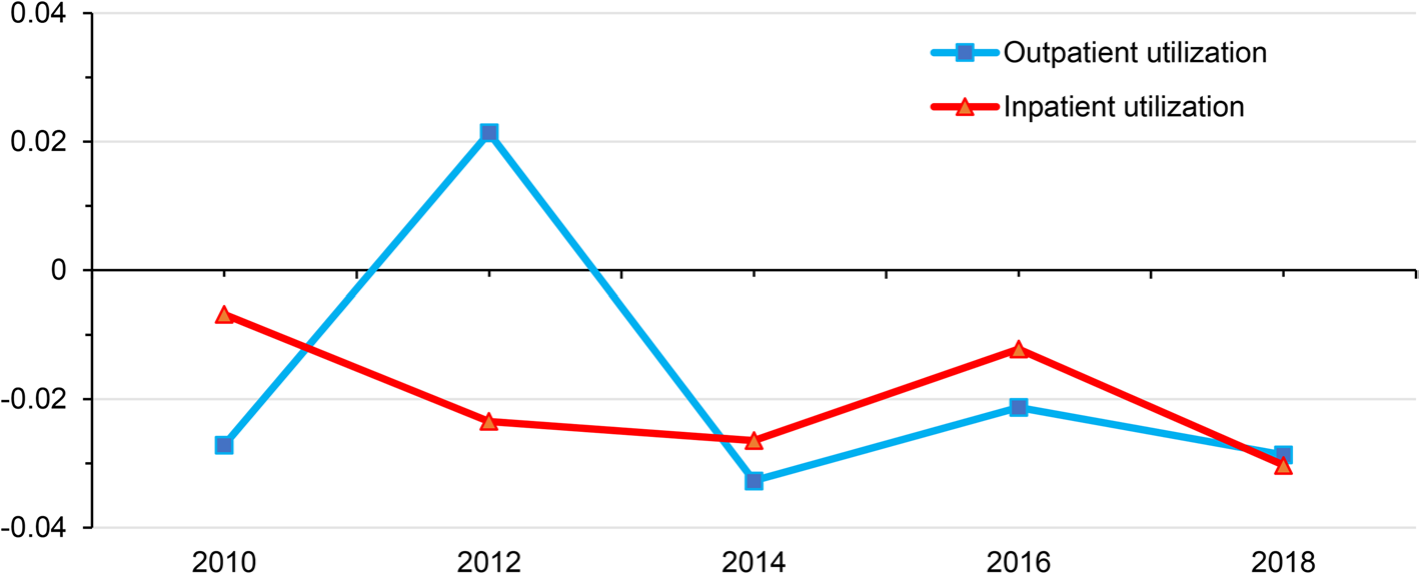
Trends in the Horizontal Inequity Index (HI), 2010–2018

In terms of the horizontal inequality index, all health services had a negative HI in all years except for outpatient utilization in 2012. The HI utilized for outpatient services changed from -0.0272 in 2010 to -0.0287 in 2018, and the HI utilized for inpatient services changed from -0.0068 in 2010 to -0.0303 in 2018. As seen in Fig. 3, the HI for outpatient and inpatient utilization among rural residents shows a constant fluctuating trend, with the outpatient HI reaching the largest and positive value in 2012 (HI = 0.0214). The inpatient HI in 2010 (HI = -0.0068) was close to zero.

### Decomposition of inequality in health care utilization

Table 3 (outpatient services) and Table 4 (inpatient services) show the results of the CI decomposition analysis. The first column shows the partial impact of each variable on health care utilization, the second column shows the absolute contribution of each factor to the overall CI, and the third column shows the percentage contribution of each factor. In terms of outpatient utilization, there was a significant relationship between outpatient utilization and age, gender, health status, and chronic disease prevalence (*p* < 0.05). In rural areas, individuals who were male, elderly, unhealthy, and with chronic disease were more likely to use outpatient services. In terms of inpatient utilization, gender did not affect the inpatient utilization of rural residents, while individuals who were elderly, unhealthy, and chronically ill were more likely to use inpatient services. There was a significant positive correlation between inpatient utilization and having a spouse, having health insurance, and being unemployed.

**Table 3:** Decomposition of inequality in outpatient utilization, 2010–2018

	**2010**			**2012**			**2014**			**2016**			**2018**		
	**dy/dx**	**Cont**	**%**	**dy/dx**	**Cont**	**%**	**dy/dx**	**Cont**	**%**	**dy/dx**	**Cont**	**%**	**dy/dx**	**Cont**	**%**
**Need factors**															
Female	-0.0249^***^	-0.0007	0.93	-0.0342^***^	-0.0011	-4.85	-0.0478^***^	-0.0004	0.53	-0.0378^***^	-0.0008	1.02	-0.0578^***^	-0.0017	1.99
< 30	-0.0506^***^	-0.0023	3.11	-0.0706^***^	-0.0062	28.32	-0.0899^***^	-0.0053	7.12	-0.0632^***^	-0.0054	7.28	-0.0822^***^	-0.0068	7.76
30 ~ 59	-0.0019	-0.0001	0.19	-0.0304^***^	-0.0022	10.07	-0.0306^***^	-0.0025	3.35	-0.0359^***^	-0.0030	4.14	-0.0343^***^	-0.0028	3.24
Poor	0.2999^***^	-0.0429	58.32	0.3038^***^	-0.0313	142.83	0.2659^***^	-0.0301	40.53	0.2707^***^	-0.0333	45.29	0.2831^***^	-0.0350	39.75
Fair	0.1412^***^	0.0057	-7.79	0.1237^***^	-0.0012	5.50	0.1115^***^	0.0008	-1.09	0.1099^***^	-0.0014	1.94	0.1079^***^	-0.0015	1.69
With chronic diseases	0.1279^***^	-0.0061	8.27	0.0836	-0.0013	6.01	0.1960^***^	-0.0041	5.50	0.1898^***^	-0.0083	11.32	0.1924^***^	-0.0114	12.94
**Non-need factors**															
Primary school or below	0.0564	-0.0140	18.99	-0.0166	0.0041	-18.80	0.0373	-0.0078	10.54	0.0389^*^	-0.0098	13.34	0.0868^***^	-0.0208	23.67
Junior high school	0.0553	0.0075	-10.27	-0.0263	-0.0030	13.65	0.0297	0.0031	-4.23	0.0135	0.0014	-1.87	0.0645^**^	0.0060	-6.77
High/Secondary school	0.0486	0.0038	-5.13	-0.0079	-0.0006	2.86	0.0318	0.0020	-2.64	0.0037	0.0003	-0.39	0.0329	0.0024	-2.70
Married	0.0063	0.0001	-0.18	-0.0078	0.0000	0.06	0.0136	0.0001	-0.16	0.0057	0.0000	0.03	-0.0147	0.0001	-0.16
With medical insurance	-0.0042	-0.0001	0.11	0.0326^**^	-0.0008	3.77	0.0177	-0.0001	0.08	-0.0023	0.0000	-0.03	0.0189	0.0000	0.03
Employed	0.0058	0.0007	-0.92	0.0194^**^	0.0044	-20.16	0.0013	0.0001	-0.07	0.0002	0.0000	-0.01	0.0148	0.0009	-0.99
Poorest SES	0.0134	-0.0104	14.17	-0.0335^**^	0.0243	-110.98	0.0152	-0.0099	13.32	-0.0052	0.0034	-4.66	0.0079	-0.0046	5.20
2th SES	0.0245^**^	-0.0095	12.96	-0.0169	0.0062	-28.09	0.0295^**^	-0.0096	12.90	0.0000	0.0000	0.00	0.0095	-0.0027	3.08
Middle SES	0.0013	0.0000	0.00	-0.0137	0.0000	0.01	0.0239^*^	0.0000	-0.02	0.0052	0.0000	-0.01	-0.0011	0.0000	0.00
4th SES	0.0162	0.0063	-8.55	-0.0070	-0.0026	11.68	0.0086	0.0028	-3.77	-0.0141	-0.0047	6.33	0.0078	0.0023	-2.57

*dy/dx* Marginal effects, ***Cont*** Contribution to the overall concentration index, % Contribution rate

***P-***value: ^*^0.01 ≤ *p* < 0.05; ^**^0.001 ≤ *p* < 0.01; ^***^p < 0.001

**Table 4:** Decomposition of inequality in inpatient utilization, 2010–2018

	**2010**			**2012**			**2014**			**2016**			**2018**		
	**dy/dx**	**Cont**	**%**	**dy/dx**	**Cont**	**%**	**dy/dx**	**Cont**	**%**	**dy/dx**	**Cont**	**%**	**dy/dx**	**Cont**	**%**
**Need factors**															
Female	-0.0073	-0.0005	1.00	-0.0076	-0.0006	1.11	0.0035	0.0001	-0.12	0.0017	0.0001	-0.09	0.0071	0.0005	-0.51
< 30	0.0047	0.0005	-1.11	0.0206^*^	0.0042	-8.10	0.0055	0.0007	-1.17	-0.0600^***^	-0.0103	14.89	-0.0472^***^	-0.0077	8.69
30 ~ 59	-0.0148^**^	-0.0027	5.66	-0.0156^**^	-0.0028	5.34	-0.0334^***^	-0.0056	9.02	-0.0437^***^	-0.0077	11.07	-0.0432^***^	-0.0069	7.79
Poor	0.0982^***^	-0.0355	74.31	0.1132^***^	-0.0261	50.33	0.1104^***^	-0.0265	42.92	0.1155^***^	-0.0284	41.07	0.1228^***^	-0.0289	32.57
Fair	0.0359^***^	0.0037	-7.66	0.0484^***^	-0.0014	2.71	0.0422^***^	0.0007	-1.06	0.0437^***^	-0.0011	1.59	0.0500^***^	-0.0014	1.63
With chronic diseases	0.0509^***^	-0.0065	13.53	0.0697^***^	-0.0017	3.29	0.1019^***^	-0.0047	7.60	0.1103^***^	-0.0096	13.82	0.1210^***^	-0.0140	15.72
**Non-need factors**															
Primary school or below	0.0013	-0.0008	1.77	-0.0054	0.0032	-6.14	-0.0085	0.0037	-5.99	-0.0102	0.0051	-7.43	0.0385^*^	-0.0177	19.93
Junior high school	-0.0009	-0.0003	0.69	0.0009	0.0002	-0.45	-0.0020	-0.0004	0.67	-0.0158	-0.0032	4.69	0.0194	0.0034	-3.83
High/Secondary school	0.0037	0.0007	-1.53	0.0093	0.0018	-3.40	-0.0060	-0.0008	1.28	-0.0082	-0.0013	1.85	0.0177	0.0024	-2.74
Married	0.0340^***^	0.0017	-3.49	0.0283^***^	0.0003	-0.52	0.0508^***^	0.0005	-0.88	0.0119	-0.0001	0.21	-0.0030	0.0001	-0.06
With medical insurance	0.0148^*^	0.0007	-1.50	0.0267^**^	-0.0016	3.15	0.0154	-0.0001	0.11	0.0404^***^	-0.0007	0.98	0.0630^***^	-0.0001	0.16
Employed	-0.0205^***^	-0.0060	12.56	-0.0409^***^	-0.0222	42.86	-0.0593^***^	-0.0052	8.34	-0.0443^***^	-0.0041	5.93	-0.0530^***^	-0.0061	6.82
Poorest SES	-0.0043	0.0087	-18.11	0.0017	-0.0030	5.88	0.0041	-0.0058	9.32	-0.0030	0.0040	-5.78	0.0024	-0.0026	2.96
2th SES	-0.0101	0.0101	-21.12	-0.0032	0.0028	-5.31	0.0092	-0.0064	10.44	-0.0014	0.0010	-1.38	-0.0073	0.0040	-4.53
Middle SES	-0.0115	0.0000	0.00	0.0022	0.0000	0.00	0.0040	0.0000	-0.01	-0.0060	0.0000	0.03	-0.0167	0.0000	0.01
4th SES	-0.0135^*^	-0.0136	28.44	-0.0012	-0.0010	1.94	-0.0020	-0.0014	2.27	0.0047	0.0031	-4.50	0.0021	0.0012	-1.30

*dy/dx* Marginal effects,* Cont* Contribution to the overall concentration index, % Contribution rate

*P*-value: *0.01 ≤ *p* < 0.05; **0.001 ≤ *p* < 0.01; ***p < 0.001

**Fig. 4:**
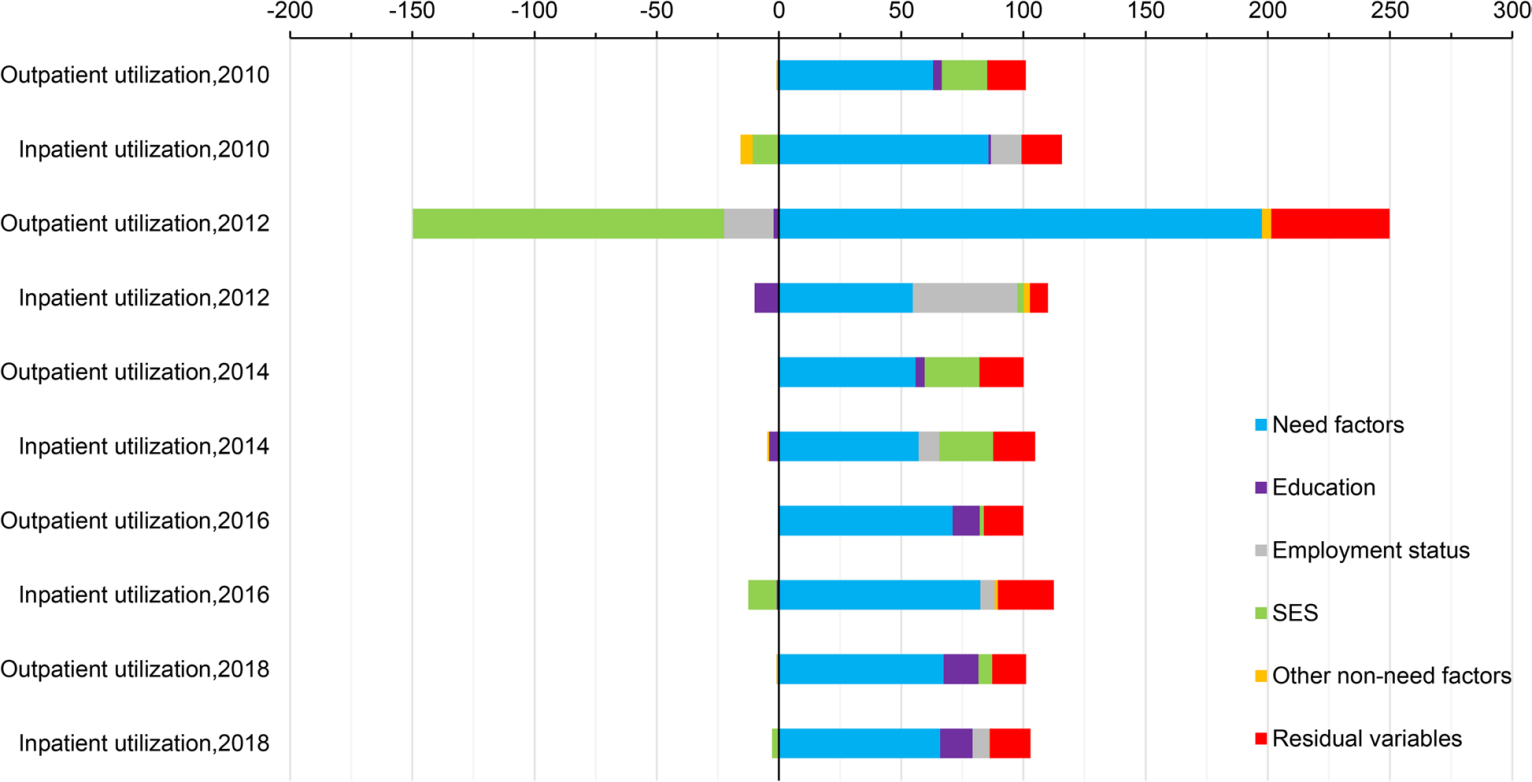
Contribution to inequality in health care utilization, China, 2010–2018 (%)

Figure 4 shows the main results of the decomposition of inequality. We found that the contribution of need factors to inequity in health care utilization among rural residents exceeded 50% in all five surveys, with a smaller proportion of inequity explained by non-need factors. The contribution of SES to inequity in outpatient utilization for rural residents was positive in all five surveys except 2012, with larger contributions in 2010 and 2014. In 2012, non-need factors, particularly the poorest SES, offset a large part of the inequity in access to outpatient services caused by need factors. In 2010, 2016, and 2018, the contribution of SES to annual inpatient utilization inequity for rural residents was negative but not significant for overall inpatient utilization inequity.

Of all the non-need factors, the larger contributions were SES, education level, and work status. Although they did not act in the same direction for overall inequitable health care utilization, the vast majority of variables contributed to inequitable rural health care utilization. Longitudinally, there was a downward trend in the contribution of work status to equity in inpatient utilization by rural residents, and it made the highest contribution to inequity in 2012. The contribution of education levels to equity in outpatient utilization among rural residents, on the other hand, showed an upward trend. In 2018, the contribution of education levels to inequitable utilization of outpatient and inpatient services reached 14.20% and 13.37%, respectively.

## Discussion

This study updates and extends the research on equity of health care utilization in rural China in three ways. Firstly, we used nationally representative longitudinal survey data from CFPS to assess health care utilization levels in rural China from 2010–2018. The findings are applicable to a wider population in China and can provide more convincing evidence on changing trends in health care utilization among rural residents. Secondly, this study also gives a comparison of the equity of health care utilization of rural residents and provides a detailed decomposition analysis of the concentration index of health care utilization of respondents, which helps to find effective ways to improve inequity. Most importantly, considering that inequity in health care utilization does not imply inequality in actual needs for health services, we measured the horizontal equity in health care utilization among rural residents, which can better reflect the impact of the Chinese government's health care reform policies aimed at increasing the accessibility of health services on residents' health care utilization since 2009.

In this study, we observed that the outpatient utilization and inpatient utilization of Chinese rural residents showed a year-to-year increase from 2010 to 2018, a phenomenon consistent with the results of the China National Health Services Survey (NHSS) [39, 40]. Since 2009, China has been carrying out the medicine and healthcare system, with the near-term goal of “effectively reducing the burden of medical expenses on residents and effectively alleviating the ‘difficulty and cost of seeing a doctor’” and the long-term goal of “establishing a sound basic medical and health care system covering urban and rural residents and providing the people with safe, effective, convenient and inexpensive medical and health care services”. Following this, the increase in the reimbursement level of the New Rural Cooperative Medical Scheme (NRCMS), the establishment of the hierarchical medical system (proposed in 2015), and family doctor services (proposed in 2016) have significantly improved the accessibility of primary health care for residents, and thus the health care need for health services for rural residents has been continuously released.

The results of this study show that the CI and HI (except for 2012) for health care utilization among rural residents from 2010–2018 were negative, which indicated that among rural populations, health care utilization remained more concentrated among low-income groups, both for outpatient and inpatient services. This result is the same as the result for equity in health services in Thailand after the implementation of the universal coverage policy [41], but the opposite of Nepal [42]. While this does not imply a corresponding improvement inequity in health outcomes, it does suggest that government policies aimed at increasing access to health services, particularly primary health care, since 2009 have helped to make health care utilization in rural China more equitable. Based on NHSS in 1993, 1998, 2003, and 2008, Zhou, Su [20] found that utilization of both outpatient and inpatient services was pro-rich in rural China with the exception of outpatient service in 2008. This study found the opposite. We speculate that this may be a result of the low level of medical coverage in rural China before 2009. At the beginning of the NRCMS, general outpatient services were not reimbursed, but only major illnesses, mainly hospitalization, were partially reimbursed [43]. Rural population was less receptive to the NRCMS and therefore although NRCMS was formally introduced in 2003, the system was in a consistent stage of expansion of the insured population until 2009. The rural residents did not fully enjoy the benefits brought by NRCMS. Most medical expenses had to be paid out of pocket, and rural residents faced a greater financial burden of illness. It is therefore reasonable that the richer groups made use of more health services. Since 2009, China has gradually introduced a dual compensation model of “inpatient coordination & outpatient coordination” in addition to the “major disease coordination & outpatient family account” compensation model. This measure amounted to a reduction in the price of outpatient and inpatient services. It reduced the financial burden on rural residents arising from outpatient and inpatient treatment, and to some extent stimulated the needs for medical services among low-income people. SES was less restrictive on the use of health services by the rural population, leading to an increase in the use of health services by the rural low-income group [44, 45].

Longitudinally, the concentration index of outpatient utilization over the years did not show a trend change. In contrast, the inequity in the use of inpatient services among rural residents was increasing, showing an increasingly pro-poor orientation, which is at odds with the findings of the majority of previous studies [46, 47]. It is well known that the price of inpatient services is much higher than the price of outpatient services, making it easier for low-income residents to fall into poverty. This phenomenon was particularly evident before the reform of medicine and healthcare system, where low-income groups were often afraid to be hospitalized to prevent the heavy medical burden associated with hospitalization. After the implementation of the reform in 2009, the inpatient compensation ratio and the ceiling line of NRCMS were raised. With a stable reimbursement rate of approximately 75% for inpatient medical expenses under NRCMS after 2012, rural residents could easily and inexpensively enjoy high-quality inpatient services, thus enhancing the utilization of inpatient services for rural low-income groups [48]. This is also consistent with the NRCMS policy goal of promoting the utilization of inpatient services by the rural population and preventing catastrophic medical expenditures [49].

Many studies have confirmed that several demographic or socioeconomic factors can influence people’s use of health services. In our study, we found that age, chronic disease, and health status showed significant positive correlations with health care utilization, and the CI decomposition results also indicated that the need variables were positively elastic to CI, suggesting that the contribution of need factors led to higher health care utilization occurring among low-income rural older people. This pro-poor inequality in health services is largely due to the unequal distribution of need factors, and income-related inequalities in health care utilization in favor of the poor were largely due to the increased demand for health care from low-income groups. This result is similar to the findings of a number of studies [50, 51]. This is not difficult to understand. As people age, their physical functions and health status decline, They need more health services [52, 53], suggesting that we should pay attention to improving the health of people with low incomes.

Among the non-need factors, education level, employment status, and SES were important explanatory factors. In most cases, employed rural residents made greater use of health services, and this was more evident in the use of inpatient services. Although this situation had gradually improved over the years, we need to pay attention to the health care needs and utilization of the unemployed or jobless in the future to guarantee their access to health services. Observing the contribution of education level to the inequitable use of outpatient services. People with lower levels of education are less well-off and do not have a higher level of health awareness [54]. Over time, an increasing number of people with low education levels had used outpatient services, suggesting that China’s health care reform policies had shown stronger policy benefits for people with low education levels. Therefore, health policymakers should consider key factors affecting equity when allocating health care resources and developing relevant interventions to meet the different health care needs of different populations.

It is worth noting that in 2012, there was a significant increase in CI utilization for rural outpatient health services and a significant decrease in CI for inpatient services. This shows that low-income people in rural areas were using more inpatient services compared to the rich and that outpatient utilization, although still pro-poor, had become much more equitable than in other years. This is consistent with the result of a study conducted by Pan [55]. In 2012, China’s NRCMS continued to focus more on the inpatient reimbursement level, with the inpatient coordinating fund accounting for more than 60% of the total annual fund financing and the reimbursement rate for rural residents’ inpatient expenses at more than 75%, while the reimbursement rate for outpatient medical expenses was less than 50%. Outpatient reimbursement levels were significantly lower than inpatient reimbursement. As a result, rural residents, especially the poor, were more likely to be hospitalized than outpatients when they were ill [56]. After 2012, China started to explore the reform of payment methods such as global budget, capitation, average cost of beds based payment, fee-for-service, and diagnosis related groups in NRCMS to promote the rational use of health resources. Based on this, doctors had changed the way they used to admit patients, with mild patients being settled in outpatient clinics and only serious patients being admitted by doctors for inpatient treatment. At the same time, with the increase in funding capacity, the reimbursement level of the NRCMS for outpatient services had also been raised. The reimbursement rate for outpatient expenses generally increased to approximately 50% and thus the use of outpatient services for low-income groups beginning to increase. After 2014, the Urban–Rural Residents Basic Medical Insurance, which combined the Urban Resident Basic Medical Insurance and NRCMS, was introduced in some regions on a trial basis and was generally implemented in 2016. Rural residents could enjoy the same medical treatment as urban residents. The level of the benefits package and reimbursement rate was standardized, and all increased, thus reducing inequities in health service utilization between different income groups [25, 57]. Of course, there is no denying that other factors, such as the hierarchical medical system, also play an important role.

Some limitations of our study must be acknowledged. The research data were collected via a self-reported questionnaire, so there may be recall bias, but this is unavoidable in all questionnaire research. In terms of variable selection, there is currently no uniform standard for the definition of need variables. In addition to variables such as age, gender, self-rated health, and chronic diseases, other variables may also be included. Therefore, there will be some bias in studying the impact of need variables on equity in health care utilization. However, at present, most scholars use these variables to represent need variables [58, 59]. Another potential limitation is the fact that we used per capita annual household income to measure the individual socioeconomic status. This choice of this measure may have underestimated the extent of extreme wealth present in rural China, although this is a common limitation of many national household surveys [60].

## Conclusions

Our study shows that after 2010, there was a significant increase in the utilization of outpatient and inpatient services by China’s rural residents. Considering the same health care needs, poor residents made use of more health services than the wealthy. The only exception was that in 2012, the rich used more outpatient services than poor rural residents. Longitudinally, the pro-poor orientation of inpatient service utilization in rural China gradually increased from 2010 to 2018. Changes in inequity in outpatient utilization were not significant. Among the non-need factors, education level, employment status and socioeconomic status were important explanatory factors. Shifting health reform policies can explain the changes in health care utilization. Since 2009, the Chinese government’s health reform policies aimed at increasing access to health services had probably been an important factor in promoting equity in health care utilization, but attention also needs to be paid to the possible unnecessary overutilization of health services. In response to the different contributions of each factor, there is still a need to design better health policies targeted at vulnerable groups and to reallocate resources to reduce inequities in the future use of health services by China’s rural population.

## Data Availability

The data were released to the researchers without access to any personal data. Data access link: http://www.isss.pku.edu.cn/cfps/en/index.htm.

## Abbreviations

CFPS: China Family Panel Study
SES: Socioeconomic Status
CI: Concentration Index
CC: Concentration Curve
HI: Horizontal Inequity index
NRCMS: New Rural Cooperative Medical Scheme
NHSS: National Health Services Survey
